# *Paraphoma chrysanthemicola* Affects the Carbohydrate and Lobetyolin Metabolism Regulated by Salicylic Acid in the Soilless Cultivation of *Codonopsis pilosula*

**DOI:** 10.3390/biology13060408

**Published:** 2024-06-03

**Authors:** Wenbin Sun, Caiming Luo, Yamiao Wu, Miao Ding, Min Feng, Feifan Leng, Yonggang Wang

**Affiliations:** School of Life Science and Engineering, Lanzhou University of Technology, Lanzhou 730050, China; sunwb@lut.edu.cn (W.S.); 232086001017@lut.edu.cn (C.L.); wym9785@163.com (Y.W.); 17393120631@163.com (M.D.); 212105500037@lut.edu.cn (M.F.); lff0928@sina.com (F.L.)

**Keywords:** *Codonopsis pilosula* (Franch.) Nannf., salicylic acid, *Paraphoma chrysanthemicola*

## Abstract

**Simple Summary:**

**Simple Summary:** The mutual interaction between endophytic bacteria *P. chrysanthemicola* and *C. pilosula* influences salicylic acid (SA) levels. Comparative analysis with the exogenous SA treatment group revealed that *P. chrysanthemicola* colonisation of *C. pilosula* exhibited an antagonistic effect on SA, mainly in carbohydrate metabolism. This phenomenon involved not only SA but also H_2_O_2_ and nitric oxide (NO) as signalling molecules.

**Abstract:**

*Paraphoma chrysanthemicola*, an endophytic fungus isolated from the roots of *Codonopsis pilosula*, influences salicylic acid (SA) levels. The interaction mechanism between SA and *P. chrysanthemicola* within *C. pilosula* remains elusive. To elucidate this, an experiment was conducted with four treatments: sterile water (CK), *P. chrysanthemicola* (FG), SA, and a combination of *P. chrysanthemicola* with salicylic acid (FG+SA). Results indicated that *P. chrysanthemicola* enhanced plant growth and counteracted the growth inhibition caused by exogenous SA. Physiological analysis showed that *P. chrysanthemicola* reduced carbohydrate content and enzymatic activity in *C. pilosula* without affecting total chlorophyll concentration and attenuated the increase in these parameters induced by exogenous SA. Secondary metabolite profiling showed a decrease in soluble proteins and lobetyolin levels in the FG group, whereas SA treatment led to an increase. Both *P. chrysanthemicola* and SA treatments decreased antioxidase-like activity. Notably, the FG group exhibited higher nitric oxide (NO) levels, and the SA group exhibited higher hydrogen peroxide (H_2_O_2_) levels in the stems. This study elucidated the intricate context of the symbiotic dynamics between the plant species *P. chrysanthemicola* and *C. pilosula*, where an antagonistic interaction involving salicylic acid was prominently observed. This antagonism was observed in the equilibrium between carbohydrate metabolism and secondary metabolism. This equilibrium had the potential to engage reactive oxygen species (ROS) and nitric oxide (NO).

## 1. Introduction

Endophytic fungi are highly esteemed for their affluent reservoir of natural bioactive compounds, endowing them with considerable value as resources [1]. Extensive studies have been conducted on the beneficial effects of endophytic fungi on host plant development and growth, which they achieve through hormone regulation [2]. It is noteworthy that endophyte–plant interactions exert their influence on plants through a range of hormones including auxins, gibberellins, cytokinins, abscisic acid, ethylene, salicylic acid, jasmonates, brassinosteroids, and strigolactone [3]. These hormones play crucial roles in promoting plant growth, facilitating nutrient absorption, enhancing stress resistance, and modulating host metabolism and immune responses [4]. Carbohydrate metabolism is a fundamental process in plant growth, development, nutrient absorption, stress resistance, and immune responses [5]. The metabolites involved in the interaction between endophytic fungi and plant carbohydrate metabolism are primarily sugars and secondary metabolites [6]. Furthermore, it has been demonstrated that endophytic fungi can stimulate the expression of specific genes in medicinal plants, ultimately activating distinct secondary metabolic pathways that lead to the accumulation of bioactive compounds [7].

In plants, the activation of cell surface receptors by endophytic fungi infection influences the signalling molecules salicylic acid (SA), hydrogen peroxide (H_2_O_2_), and nitric oxide (NO) [8,9]. SA plays a pivotal role in regulating the growth–differentiation balance in plants, influencing plant immunity and growth [10]. It serves as a mediator in plant defence responses to biotic and abiotic stresses, as well as influencing various processes, including seed germination, seedling growth, total chlorophyll concentration, nutrient uptake and transport, flowering, metabolism, expression of aging-related genes, induction of antioxidant defence systems, and pathogen resistance [11,12]. The invasion of endophytic fungi into plants has the potential to disrupt the plant’s carbohydrate metabolism, which can be further modulated by salicylic acid’s regulation of the immune system [13]. The symbiotic relationship between fungi and plants enhances carbohydrate levels, including glucose, sucrose, and starch, through processes such as the glycolysis pathway [14,15]. Conversely, other endophytes inhibit the carbohydrate metabolism of plants [16,17]. This is achieved by regulating key enzymes involved in sucrose metabolism, including invertase and sucrose synthase, thereby providing essential energy and carbon sources for secondary metabolism [18,19]. Lobetyolin, a vital secondary metabolite in *Codonopsis pilosula* (Franch.) Nannf., exhibits a positive correlation with the diversity of its resident communities of endophytes, as supported by empirical research [20].

*C*. *pilosula*, a member of the *Campanulaceae* family, is widely used for its medicinal properties. The dried root of *C. pilosula* is commonly employed as a medicine due to its high medicinal value [21,22,23]. Recent studies have focused on the root of *C. pilosula*, revealing its potential to lower blood pressure [24], improve memory impairment [25], regulate immune function [26], and exhibit antioxidant and anti-aging effects [27]. In addition, the stem, leaf, and root of *C. pilosula* possess antioxidant and antimicrobial activities [28,29,30]. *C*. *pilosula* is characterised by an abundant endophytic bacterial community residing in its tissues [20]. *Paraphoma chrysanthemicola* was first described in the *Phoma* section *Peyronellaea* [31]. *P. chrysanthemicola* is a pathogenic fungus, but as a symbiont in *C. pilosula* it does not cause disease in the host plant [32]. In this paper, the endophytic fungus *P. chrysanthemicola* sp. DS-84 was isolated from *C. pilosula* roots in our previous experimental study [33]. However, the specific interaction between SA and endophytic fungi and its effects on plants such as *C. pilosula*, remains uncertain. Therefore, this study aims to investigate the influence of *P. chrysanthemicola* on the growth and accumulation of compounds in *C. pilosula* under the influence of SA.

## 2. Materials and Methods

### 2.1. Seed Treatments and Strain Preservation

*C. pilosula* was collected from Lintao County, Dingxi City, Gansu Province, China (103°29′08″–104°19′34″ E, 35°03′42″–35°56′46″ N) and certified by Professor Lin Yang (Department of Pharmaceutical Engineering, School of Life Science and Engineering, Lanzhou University of Technology). The region is characterised by a temperate continental climate, with an average annual temperature of 7 °C (maximum 34.6 °C, minimum −29.5 °C), an average annual precipitation of 317–760 mm, and an evaporation of more than 1400 mm.

In this study, the seeds of *C. pilosula* were washed with sterile water and soaked in 75% ethanol for 10 s. Subsequently, the seeds were immersed in 2% sodium hypochlorite for two minutes and then rinsed thoroughly with sterile water. Subsequently, the seeds were stored at room temperature following a 12 h soak in sterile water.

The fungal strain was named *P. chrysanthemicola* DS-84. The genome accession was JARVTL000000000.1. The strain was previously isolated from the roots of *C. pilosula*. The strain has been preserved in the laboratory using an oblique surface and glycerol methods [33].

### 2.2. Co-Cultivation between Fungus and Plants

The vermiculite was initially sterilised using high-temperature steam pressure sterilisation and placed in a square plastic pot measuring 10 cm × 10 cm with a capacity of 220 mL. The seeds were then evenly distributed in the vermiculite and grown in a greenhouse maintained at a constant temperature and humidity. The water content of the pot was measured twice daily, in the morning and evening. The seeds were irrigated on a regular basis using a modified Hogland nutrient solution with the following concentrations: FeSO_4_·7H_2_O, 20 mM; EDTA, 22 mM; Ca(NO_3_)_2_·4H_2_O, 4 mM; KNO_3_, 5 mM; MgSO_4_, 4 mM; NH_4_NO_3_, 1 mM; KH_2_PO_3_, 1 mM; MnSO_4_, 0.15 μM; H_3_BO_3_, 0.1 μM; ZnSO_4_, 0.05 μM. Additionally, the solution contained KI at a concentration of 5 μM and the following micronutrients: Na_2_MoO_4_, CoCl_2_, and CuSO_4_, at concentrations of 0.2 μM, 0.15 μM, and 0.15 μM, respectively.

Three weeks after seed germination, 10 plants of *C. pilosula* seedlings with uniform growth status were retained in each pot. After that, seedlings were treated with sterile water (CK, 30 mL), *P. chrysanthemicola* solution (FG, 10^5^ colony-forming units per milliliter, 30 mL), salicylic acid (SA, 0.2 mM, 30 mL), *P. chrysanthemicola* and salicylic acid mixed solution (FG+SA, 10^5^ colony-forming units per milliliter + 0.2 mM, 30 mL), respectively. Among the treatments, the concentration of SA depends on previous experiments [34,35,36]. The concentration and amount of inoculum were obtained from a previous experiment of our group [37]. After 45 days of co-cultivation, the plants with complete parts and consistent growth status were selected, and their roots, stems, and leaves were taken for sampling and measurement and repeated 6 times per group.

### 2.3. Determination of Morphological Indicators

Fresh plant samples were collected in order to determine the upper plant height, fresh weight, and number of leaves of *C. pilosula* seedlings. The plant roots were washed with water, and then rinsed with deionized water. The surface water droplets were absorbed with filter paper in order to measure the fresh weight, diameter, and length of the root. Four to five leaves were collected from each plant and the leaf area was calculated using ImageJ software (version 2022, National Institutes of Health, Bethesda, MD, USA). Fresh root, stem and leaf samples were collected and immediately flash-frozen in liquid nitrogen and stored at −80 °C.

### 2.4. Detection of Chlorophyll and Carbohydrate Content

The chlorophyll (Chl) content was determined using a colorimetric method for acetone extraction [38]. A total of 0.2 g of leaf tissue was randomly weighed and repeated six times per group. The Chl was extracted from the leaves of *C. pilosula* seedlings in a solution of acetone/water (4/1, *v*/*v*). The absorbance of the extracts was recorded using an ultraviolet spectrophotometer (Lonico Instrument Co., Ltd., Shanghai, China) at 645 and 663 nm.

The soluble sugar content was quantified using the phenol sulfuric acid (PSA) method [39]. *C. pilosula* seedlings were divided into three parts: stem, leaf, and root. In subsequent methods presented in this paper, the three parts of the *C. pilosula* seedlings were analysed separately. Specifically, 0.1 g of each part was weighed and repeated six times per group. A total of 500 μL of phenol solution (50 g·L^−1^) and 2.5 mL of concentrated sulfuric acid were added successively and mixed evenly. After cooling to room temperature, the soluble sugar content was read on an ultraviolet spectrophotometer (Lonico Instrument Co., Ltd., Shanghai, China) at an absorbance of 490 nm.

The reduced sugar content was determined using 3,5-dinitrosalicylic acid (DNS) [40]. A 0.1 g of sample was weighed and 1 mL of the DNS solution was added, followed by heating in boiling water for 15 min. The colour intensity of the solution was then measured at 540 nm using an ultraviolet spectrophotometer (Lonico Instrument Co., Ltd., Shanghai, China). The concentration of reduced sugar was calculated using a calibration curve of a standard glucose solution [41]. The polysaccharide content was determined by subtracting the concentration of reduced sugars from the total sugar concentration.

Ultraviolet-based high-performance liquid chromatography (HPLC/UV) was employed to ascertain the fructose, glucose, and sucrose content [42]. A stock solution (10 mL) comprising 100 mg each of fructose, glucose, and sucrose was prepared. Standard solutions were generated from the stock solution by twofold serial dilution (10–0.039 mg·mL^−1^) to construct a calibration curve. Samples from *C. pilosula* seedlings were crushed, diluted in 100 μL of water, and then made into a 10 mL solution (100-fold dilution). The wavelength range of HPLC (JASCO LC, Seltai Technology Co., Ltd., Hangzhou, China) was adjusted from 190 nm to 400 nm.

### 2.5. Detection of Soluble Protein and Lobetyolin Content

The Coomassie brilliant blue G-250 method was employed to determine the soluble protein content of the solution by using a visible spectrophotometer (V-5100B, Shanghai Yuanyin Instrument Co., Ltd., Shanghai, China). The wavelength at which the detection occurred was 595 nm [43].

The lobetyolin content was quantified using HPLC/UV, following the method described in the report by He [44]. Dried and powdered *C. pilosula* (1.0 kg) was extracted by steeping in methanol three times (10 L, 48 h) at room temperature. The combined methanol extracts were evaporated under reduced pressure and then lyophilized to give a brown solid residue. The residue was redissolved in distilled water and applied to a column (9 cm × 55 cm) for elution with the following solvents: H_2_O, 20%, 40%, 60%, and 80% methanol (5.0 L of each solvent). After detection of each eluent by thin layer chromatography (TLC), the eluents with similar chemical compositions were combined and dried by reduced evaporation and lyophilization.

### 2.6. Analysis of Endogenous Signalling Molecules

The presence of NO was determined using the fluorescent probe DAF-FM DA [45]. In brief, 10 μL DAF-FM DA (10 μM) was applied to the slide, and the tissues of *C. pilosula* were placed on the coverslips. Subsequently, the samples were incubated at 37 °C for 30 min and washed with 10 mM phosphate buffer saline (PBS) to remove residual dye solution. Finally, the NO fluorescence was observed under a fluorescence microscope (XK-DL005, Sinico Optical Instrument Co., Ltd., Shenzhen, China).

SA was identified by HPLC [46]. The samples were ground to homogenize them and then immersed in 90% methanol (20 mL). The homogenate was then centrifuged for 30 min at 10,000 r·min^−1^. The supernatant was removed and dried with a rotary evaporator (Seltai Technology Co., Ltd., Hangzhou, China). The solution was dissolved twice with 5% trichloroacetic acid (15 mL), and a 40 mL mixture of ethyl acetate and cyclohexane was added. The liquid phase consisted of 80% methanol and 20% sodium acetate (0.2 mol·L^−1^, pH 5.5, 30 mL).

JA was extracted and determined by HPLC according to Liu [47]. Following the grinding of 500 mg of fresh *C. pilosula* in liquid nitrogen, 600 μL of methanol (80%) was added, mixed, and then stored overnight at 4 °C. Samples were centrifuged at 4 °C (10,000 r·min^−1^, 10 min). The supernatant was transferred to a new tube, extracted with 200 μL methanol (80%), dried, and then dissolved in 0.2 mol·L^−1^ Na_2_HPO_4_-H_3_PO_4_ buffer and extracted three times with petroleum ether until the upper part was colourless. The pH was adjusted to 8.0 and extracted three times with ethyl acetate. Samples were detected at the wavelength of 195 nm.

H_2_O_2_ content was quantified in accordance with previous research [48]. Five millilitres of precooled acetone was added to 0.2 g of fresh material and rapidly homogenized in an ice bath. The homogenate was then centrifuged at 4 °C (10,000 r·min^−1^, 10 min). Two millilitres of the supernatant was taken and mixed with 5% titanium sulfate (0.5 mL) and 2 mL of concentrated ammonia water. The solution was centrifuged at 4 °C (10,000 r·min^−1^, 10 min) and the supernatant was discarded. The precipitate was dissolved with 5 mL concentrated sulfuric acid (2 mol·L^−1^) and then detected at 415 nm.

### 2.7. Detection of Viability of the Antioxidant Enzyme Lines and Detection of Metabolite Enzyme Lines Related to Metabolite Synthesis

Tissue samples (45 days) of roots, stems, and leaves (0.6 g of each) were ground with 0.05 mol·L^−1^ phosphate buffer (pH 7.8) in an ice-cold mortar. The homogenates were centrifuged at 4 °C (12,000× *g*, 10 min), and the supernatant was collected for activity analysis. The activities of catalase (APX), peroxidase (SOD), superoxide dismutase (POD), ascorbate peroxidase (CAT), and sucrose synthase (SS) were determined using enzyme activity kits (Suzhou Keming Biotechnology Co., Ltd., China). The above measurements were repeated 6 times for each group.

The activity of APX (EC 1.11.1.11) was calculated by measuring the oxidation rate of ascorbate acid (AsA) at 290 nm, with H_2_O_2_ serving as the control. The absorbance change of the reaction mixture was measured at 290 nm for 10 s and 130 s, designated as A1 and A2, respectively. An enzyme activity unit was defined as the oxidation of 1 nmol AsA per gram of tissue per minute. APX activity was calculated using the following formula: APX activity (nmol·min^−1^·g^−1^ FW) = 1786 × ∆A ÷ W (W: fresh weight of sample, g; ∆A = A1 − A2).

The activity of SOD (EC 1.15.1.1) was quantified by utilizing xanthine and xanthine oxidase (XO) to generate O_2_^−^. O_2_^−^ can reduce nitroblue tetrazole (NBT) to form blue methine, which was absorbed at 560 nm, and SOD can remove O_2_^−^ to inhibit the formation of methine. The inhibition of 50% in the reaction system was defined as an enzyme activity unit. The SOD activity was calculated using the following formula: SOD activity (U·g^−1^ FW) = 11.4 × percentage of inhibition ÷ (1 − percentage of inhibition) ÷ W (percentage of inhibition= [A (control) − A (experiment)] ÷ [A (control)] × 100%; W: fresh weight of sample, g).

POD (EC 1.11.1.7) activity was calculated according to the characteristic absorption of an aspecific substrate that catalyses H_2_O_2_ oxidation at 470 nm. The reaction mixture absorbance change was measured at 470 nm for 1 min and 2 min, designated as A1 and A2, respectively. One POD activity unit was defined as the change in absorbance of 0.01 per gram of tissue per minute at 470 nm in the reaction system. The POD activity was calculated according to the following formula: POD activity (U·g^−1^ FW) = 2000 × ∆A ÷ W (W: fresh weight of sample, g; ∆A = A2 − A1).

The activity of CAT (EC 1.11.1.6) was determined based on monitoring the absorbance of H_2_O_2_ at 240 nm. The initial absorbance (A1) of the reaction mixture at 240 nm was measured, followed by the absorbance (A2) after one minute. The difference between these two values (∆A) was calculated. One CAT activity unit was defined as the degradation of 1 nmol H_2_O_2_ per gram of tissue per minute. The CAT activity was calculated according to the following formula: CAT activity (nmol·min^−1^·g^−1^ FW) = 2000 × ∆A ÷ W (W: fresh weight of sample, g).

The activity of SS (EC 2.4.1.13) activity was determined by observing a colour change when it reacted with resorcinol and noting the characteristic absorption peak at 480 nm. One SS activity unit was defined as the amount of sucrose catalysed per gram of tissue per minute. The SS activity was calculated according to the following formula: SS activity (μg·min^−1^·g^−1^ FW) = 100 × [A (experiment) − A (control)] ÷ [A (standard) − A (blank)] ÷ W (W: fresh weight of sample, g).

Invertase (Ivr) is a catalyst that irreversibly decomposes sucrose into fructose and glucose. In accordance with the optimal pH, Ivr can be classified into acid invertase (AI) and neutral invertase (NI). Soluble acid invertase (S-AI) and cell wall-insoluble acid invertase (B-AI) are two forms of acid invertase (AI). In the experiment, vacuolar acid convertase (VAIV) was identified as belonging to the S-AI category, cytoplasmic convertase (CInv) was classified as NI, and cell wall acid convertase (BAIV) was designated as B-AI. Tissue samples (45 days) of roots, stems, and leaves (0.3 g of each) were ground with 0.05 mol·L^−1^ phosphate buffer (VAIV buffer pH was 4.5, CInv was 7.0, BAIV was 7.8) in an ice-cold mortar. Centrifuge the homogenates at 4 °C (12,000 g, 10 min) and collect the supernatant for subsequent activity analysis. The activity of VAIV, CInv, and BAIV was quantified using a commercially available kit (Suzhou Keming Biotechnology Co., Ltd., Suzhou, China). The aforementioned measurements were conducted six times for each group.

The activity of the VAIV, CInv, and BAIV was determined by catalysing the reaction of sucrose with 3,5-dinitrosalicylic acid to generate brownish-red amino compounds with characteristic light absorption at 510 nm. An enzyme activity unit was defined as 1 μg of reducing sugar per minute per gram of tissue at 37 °C. The principal distinction between the VAIV, CInv, and BAIV measurement methodologies was the pH value of the buffer (VAIV buffer pH was 4.5, CInv was 7.0, BAIV was 7.8). Activity was calculated using the following formula: VAIV/CInv/BAIV activity (μg·min^−1^·g^−1^ FW) = 20.8 × (ΔA + 0.001) ÷ W [∆A = A (measurement) – A (control); W: fresh weight of sample, g].

### 2.8. Data Processing and Statistical Analysis

The raw data were statistically collated using Microsoft Excel 2010. Normal distribution test and variance homogeneity test were used for one-way analysis of variance (ANOVA), and Duncan multiple range test was used for significant difference analysis (α = 0.05). These tests were conducted in the statistical software package SPSS (version 17.0, SPSS, Inc., Chicago, IL, USA). The partial least squares regression (PLSR) analysis was performed using the pls package in the statistical programming language R (version 2.8.3). The method parameter was specified as “oscorespls”, the validation plot employed “CV”, and the remaining parameters were retained at their default settings. Structural equation models (SEM) were estimated using the seminr package in R (version 2.3.2) for partial least squares path modelling (PLS-SEM), with all parameters set to their default values. The drawing and splicing of the data were conducted using Origin (version 9.0, OriginLab, Inc., Northampton, MA, USA) and Adobe Illustrator 2022 (Adobe Systems, Inc., San Jose, CA, USA).

## 3. Results

### 3.1. Effects on C. pilosula Seedling Growth and Total Chlorophyll Concentration

**Figure 1:**
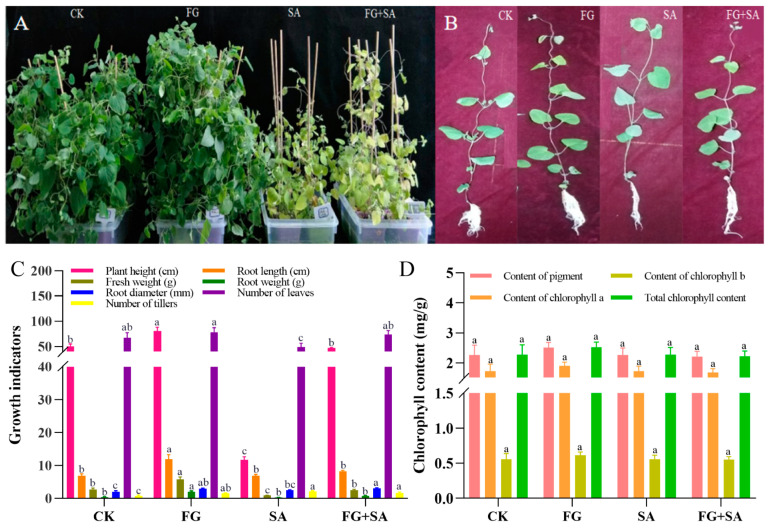
Changes in morphology and chlorophyll of *C. pilosula* under FG and SA treatments. (**A**) Growth status of plants under each treatment. (**B**) Growth status of individual plants under each treatment. (**C**) Morphological characteristics under each treatment. (**D**) Chlorophyll content under each treatment. CK: sterile water; FG: *P*. *chrysanthemicola* solution; SA: salicylic acid; FG+SA: *P. chrysanthemicola* and salicylic acid mixed solution. The height of the bar chart represents the numerical value, and a,b,c indicates the significance between treatment groups.

The findings from the 40-day treatment of *C. pilosula* revealed that the FG group exhibited the most robust growth when compared to the control group. Conversely, growth was inhibited in both the SA and SA+FG groups (Figure 1A). Detailed examination of individual plants revealed adverse effects on the growth of roots, stems, and leaves of *C. pilosula* (Figure 1B). Among all treatment groups, FG plants exhibited the greatest height (80.47 cm), root length (11.97 cm), fresh weight (5.74 g), root weight (2.05 g), and number of leaves and trillers (78; *p* < 0.05) (Figure 1C). The SA group exhibited the lowest values for plant height, fresh weight, and number of leaves among the experimental groups (11.77 cm, 0.89 g, and 49, respectively; *p* < 0.05) (Figure 1C). The values of plant height, fresh weight, root diameter, and number of leaves were found to be higher in the FG+SA group than in the SA group (47.38 cm, 2.44 g, 3.02 mm, and 71.20; *p* < 0.05) (Figure 1C). No significant difference in chlorophyll content was observed among the groups (*p* < 0.05) (Figure 1D).

### 3.2. Effects of Different Treatments on Carbohydrate Content in C. pilosula

**Figure 2:**
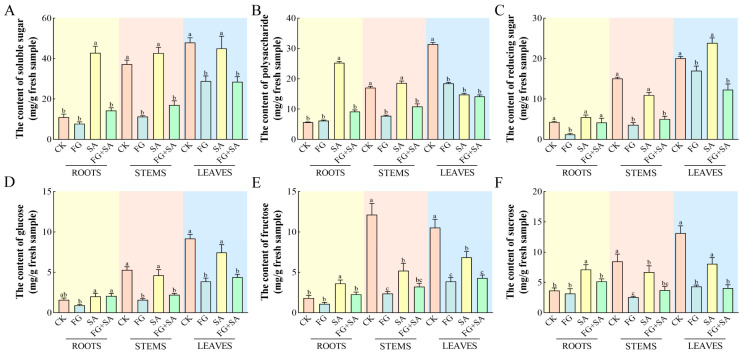
The effects of *P. chrysanthemicola* and SA on the carbohydrate contents in *C. pilosula*. The content of (**A**) soluble sugars, (**B**) polysaccharide, (**C**) reducing sugar, (**D**) glucose, (**E**) fructose, and (**F**) sucrose in roots, stems, and leaves under four treatments. CK: sterile water; FG: *P*. *chrysanthemicola;* SA: salicylic acid; FG+SA: *P. chrysanthemicola* and salicylic acid mixed solution. The height of the bar chart represents the numerical value, and a,b,c indicates the significance between treatment groups.

In comparison to the control group, the FG group exhibited significant reductions (*p* < 0.05) in the levels of all detected carbohydrates in both the stems and leaves. Furthermore, a significant reduction (*p* < 0.05) in the content of reducing sugars was observed solely in the roots (Figure 2B). The roots of the SA group exhibited the highest content of soluble sugars, polysaccharides, sucrose, and fructose, with values of 36.21, 25.32, 4.23, and 7.35 mg·g^−1^, respectively (Figure 2A,B,E,F). However, when compared to the control group, the SA group exhibited significant reductions (*p* < 0.05) in polysaccharide and fructose content in leaves, as well as fructose and sucrose content in stems (Figure 2A,E,F). The FG+SA group demonstrated a statistically significant reduction (*p* < 0.05) in soluble sugar, polysaccharide, fructose, and sucrose content in roots compared to the SA group (Figure 2A,B,E,F). The FG+SA group demonstrated a statistically significant reduction (*p* < 0.05) in soluble sugar, polysaccharide, reducing sugar, and glucose content in stems in comparison to the SA group (Figure 2A,B,C,D).

### 3.3. Effects of Different Treatments on the Accumulation of Signal Molecules in C. pilosula

**Figure 3:**
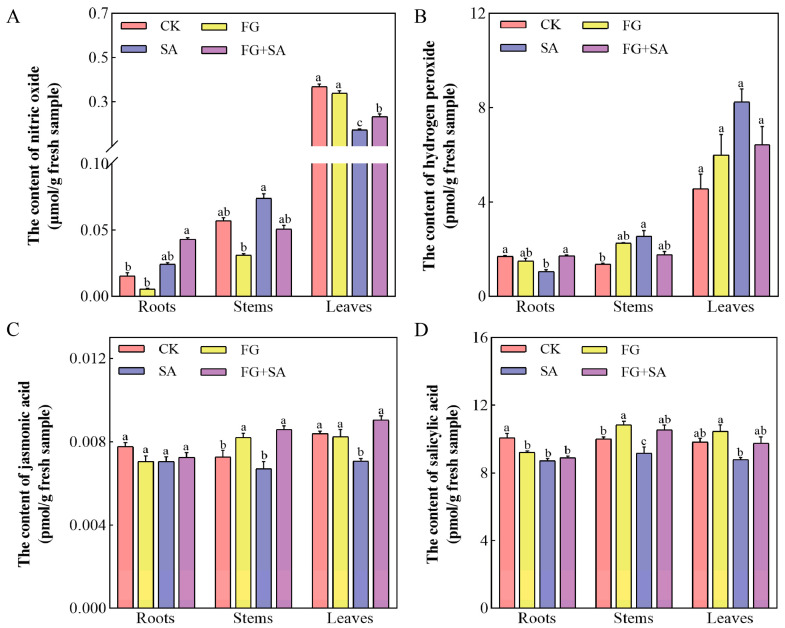
Effects of *P. chrysanthemicola* and SA on signalling molecules in *C. pilosula*. Effect of four treatment groups on (**A**) nitric oxide content, (**B**) H_2_O_2_, (**C**) JA, and (**D**) SA in roots, stems, and leaves. CK: sterile water; FG: *P*. *chrysanthemicola*; SA: salicylic acid; FG+SA: *P. chrysanthemicola* and salicylic acid mixed solution. The height of the bar chart represents the numerical value, and a,b,c indicates the significance between treatment groups.

In comparison to the control group, the FG group did not demonstrate a significant impact on the levels of NO, H_2_O_2_, and JA in the roots, stems, and leaves of the plants (Figure 3). The FG group has been shown to significantly reduce the endogenous levels of SA in the roots, while simultaneously increasing the SA content in the stems. In contrast, the SA group demonstrated a suppressive effect on NO production in leaves and H_2_O_2_ production in roots, while simultaneously inducing H_2_O_2_ production in stems (Figure 3A,B) (*p* < 0.05). In comparison to the SA group, the FG+SA group exhibited an elevation in H_2_O_2_ levels in roots, JA levels in stems, and both NO and JA levels in leaves (Figure 3A–C) (*p* < 0.05).

### 3.4. Effects on the Accumulation of Soluble Protein and Lobetyolin Content

**Figure 4:**
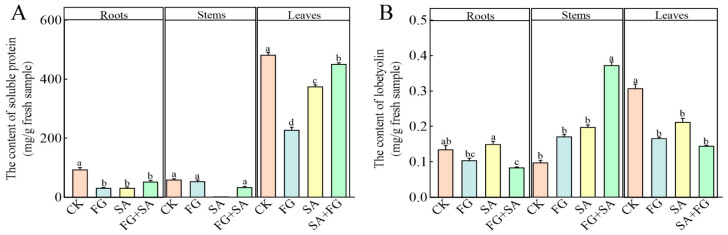
Effects of *P. chrysanthemicola* and SA on the accumulation of (**A**) soluble protein and (**B**) lobetyolin content in *C. pilosula*. CK: sterile water; FG: *P. chrysanthemicola* solution; SA: salicylic acid; FG+SA: *P. chrysanthemicola* and salicylic acid mixed solution. The height of the bar chart represents the numerical value, and a,b,c indicates the significance between treatment groups.

The FG and SA groups exhibited a notable reduction in soluble protein content in roots and leaves, when compared to the control group (*p* < 0.05) (Figure 4A). The soluble protein content in the leaves of the FG group exhibited the lowest value, with a decrease of 39.34% compared to the SA group (*p* < 0.05). The FG+SA group demonstrated a significant alleviation of the inhibitory effect in the leaves, resulting in a 20.23% increase compared to the SA group (*p* < 0.05).

In comparison to the control group, both the FG and SA treatments demonstrated a significant inhibitory effect on lobetyolin in the leaves. However, in the roots, the lobetyolin content was significantly higher in the SA group compared to the FG group, with a 43.58% increase (*p* < 0.05) (Figure 4B). Conversely, the FG+SA treatment demonstrated a significant inhibitory effect on lobetyolin content in roots, while simultaneously exhibiting a stimulatory effect on lobetyolin content in stems when compared to the SA group (*p* < 0.05).

### 3.5. Effects of Different Treatments on Antioxidase-like Activity in C. pilosula

**Figure 5:**
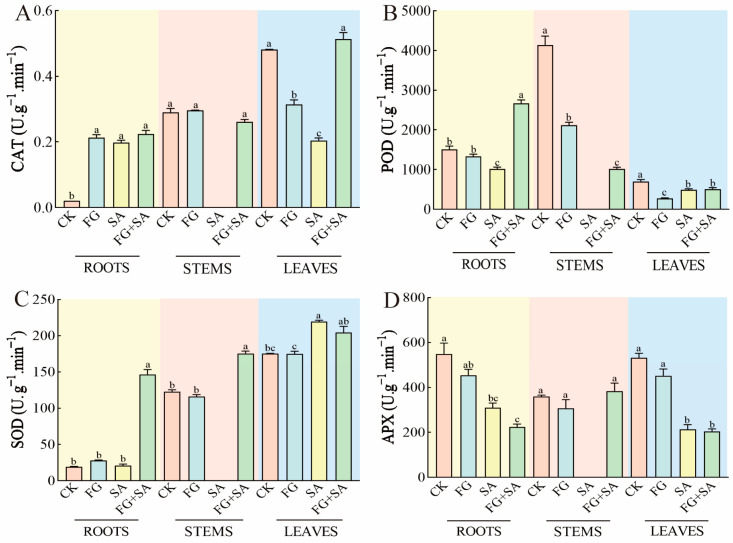
Effects of *P. chrysanthemicola* and SA on antioxidase-like activity in *C. pilosula*. The effect of *P. chrysanthemicola* and SA on (**A**) CAT activity, (**B**) POD, (**C**) SOD, and (**D**) APX. CK: sterile water; FG: *P*. *chrysanthemicola* solution; SA: salicylic acid; FG+SA: *P. chrysanthemicola* and salicylic acid mixed solution. The height of the bar chart represents the numerical value, and a,b,c indicates the significance between treatment groups.

The administration of FG resulted in an increase in CAT activity in roots, while simultaneously inhibiting CAT activity in leaves when compared to the control group. The results observed in the SA group exhibited similar patterns to those observed in the FG group, although with a more pronounced inhibition of CAT activity in leaves by SA compared to FG. In contrast, the FG+SA treatment demonstrated an increase in CAT activity in leaves when compared to the SA group (*p* < 0.05) (Figure 5A).

The activity of POD in roots exhibited a significant inhibition in FG treatment in stems and leaves (*p* < 0.05). In roots, the suppression of POD activity was more pronounced in the group treated with SA compared to the group treated with FG (decreased by 23.68%, *p* < 0.05). Conversely, the FG+SA group exhibited significantly higher POD activity in roots compared to the SA group (increased by 162.69%, *p* < 0.05) (Figure 5B).

The activity of SOD in the leaves of the group treated with SA demonstrated a notable increase of 25.36% in comparison to the control group (*p* < 0.05). A similar pattern was observed in the FG+SA group, where the activity of SOD in the roots exhibited a substantial increase of 597.53% (*p* < 0.05) compared to the SA group (Figure 5C).

In comparison to the control group, the FG treatment did not affect the APX activity. However, the SA treatment significantly inhibited APX activity in both roots (reduction of 43.67%) and leaves (reduction of 59.92%) (*p* < 0.05). Furthermore, the inhibitory effect of the FG+SA treatment on APX activity in roots was greater than that observed in the SA treatment group (reduction of 4.10%) (Figure 5D).

### 3.6. Effects on Enzymatic Activity Related to Glucose Metabolism in C. pilosula under Treatment of P. chrysanthemicola and Salicylic Acid

**Figure 6:**
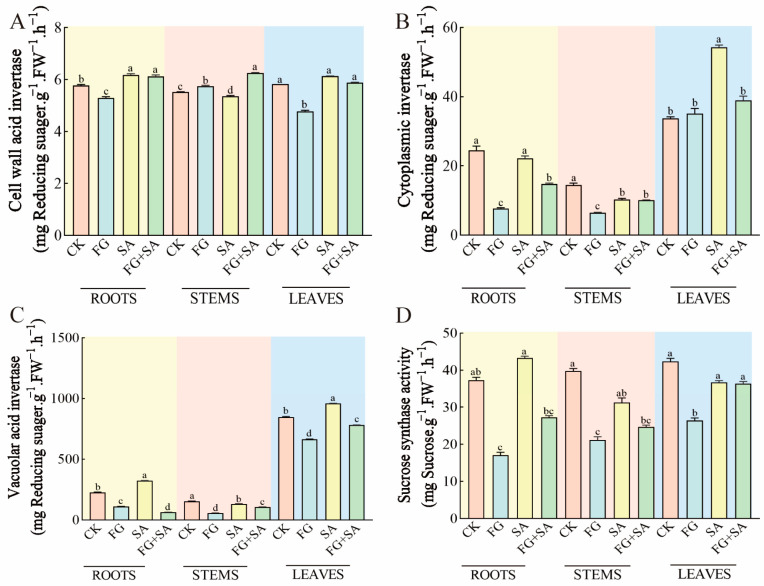
Effects of *P. chrysanthemicola* and SA on enzymatic activity related to carbohydrate metabolism in *C. pilosula*. The content of (**A**) BAIV, (**B**) CInv, (**C**) VAIV, and (**D**) SS in the roots, stems, and leaves under four different treatments. CK: sterile water; FG: *P*. *chrysanthemicola* solution; SA: salicylic acid; FG+SA: *P. chrysanthemicola* and salicylic acid mixed solution; BAIV: cell wall acid invertase; CInv: cytoplasmic invertase; VAIV: vacuolar acid invertase; SS: sucrose synthethase. The height of the bar chart represents the numerical value, and a,b,c indicates the significance between treatment groups.

The experimental results indicated that the application of FG and SA had the opposite effect. The FG group exhibited a notable decrease in BAIV content in both roots (8.50% reduction) and leaves (18.04% reduction) compared to the control group (*p* < 0.05). Conversely, the SA group exhibited a significant decrease in BAIV content in stems (2.90% decrease, *p* < 0.05) but an increase in BAIV content in roots and leaves (7.1% and 5.15% increase, *p* < 0.05). Furthermore, the FG+SA treatment resulted in an increase in BAIV content in stems (16.82% increase) compared to the SA group (*p* < 0.05) (Figure 6A).

A comparison of the control group with the FG group revealed a 68.47% and 54.80% reduction in the content of CInv in the roots and stems, respectively (*p* < 0.05). In contrast, the SA group exhibited a 28.99% decline in the content of CInv in the stems, yet a 61.18% increase in the leaves (*p* < 0.05). Furthermore, the content of CInv decreased by 33.40% in the roots and by 28.18% in the leaves in comparison to the SA group (*p* < 0.05) (Figure 6B).

In the FG treatment group, the content of VAIV decreased by 51.23%, 63.55%, and 21.67% in the roots, stems, and leaves, respectively, in comparison to the control group. Conversely, in the SA group, there was an increase of 42.32% and 13.14% in the roots and leaves, respectively (*p* < 0.05). Furthermore, in the FG+SA group, the VAIV content in the roots, stems, and leaves was significantly lower compared to the SA group, with reductions of 80.26%, 24.76%, and 18.79%, respectively (*p* < 0.05) (Figure 6C).

The FG group exhibited inhibition in the content of SS in the roots, stems, and leaves, with reductions of 52.09%, 46.25%, and 38.31%, respectively (*p* < 0.05). In contrast, no significant change in SS content was observed in the roots, stems, and leaves of the SA group. In comparison to the SA group, the FG+SA group exhibited a 36.96% decrease in SS content in the roots (*p* < 0.05) (Figure 6D).

### 3.7. Correlation Analysis of the Physiological and Biochemical Indexes of C. pilosula

**Figure 7:**
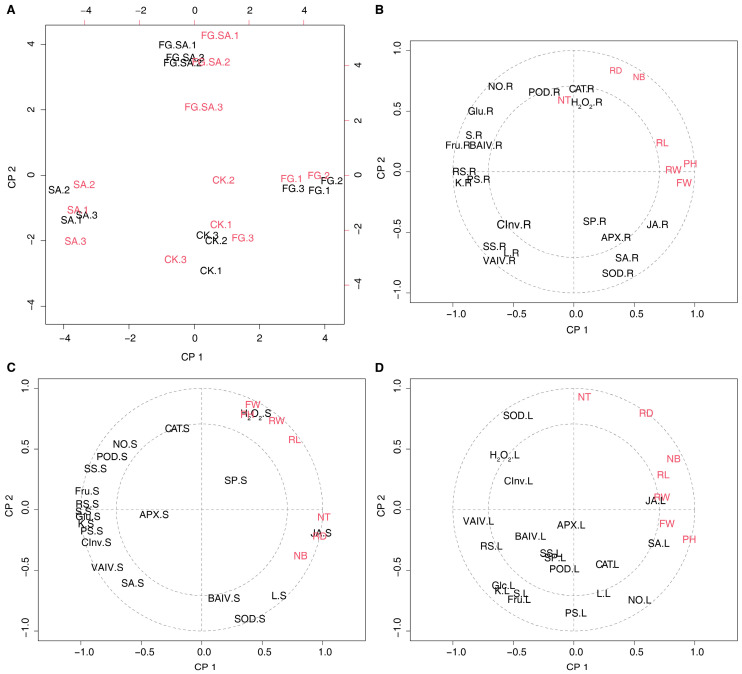
PLSR analysis of phenotypic changes versus physiological changes in *C. pilosula* in three organs. (**A**) PLSR analysis of the scores plotted in the results of phenotypic changes versus physiological changes in roots. (**B**–**D**) Correlation loadings between phenotypic changes and physiological changes in the root (**B**), stem (**C**), and leaf (**D**). PH: plant height; FW: fresh weight; RL: root length; RW: root weight; RD: root diameter; NB: the number of leaves; NT: the number of tillers; KS: soluble sugar; RS: reduced sugar; PS: polysaccharide; Glc: glucose; Suc: sucrose; Fru: fructose; SP: soluble protein; L: lobetyolin; BAIV: cell wall acid invertase; CInv: cytoplasmic invertase; VAIV: vacuolar acid invertase; SS: sucrose synthethase; NO: nitric oxide; H_2_O_2_: hydrogen peroxide; SA: salicylic acid; JA: jasmonate acid; POD: peroxidase; SOD: superoxide dismutase; CAT: catalase; APX: ascorbate peroxidase; R: root; S: stem; L: leaf. The two concentric circles are the default radii corresponding to 50% and 100% explained variance.

The correlation between carbohydrates, signalling molecules, secondary metabolites, antioxidant enzyme activity, glucose metabolism-related enzyme activity, and phenotypes was further analysed using PLSR. The calculations performed for each of the three tissues demonstrated opposing results between the FG and SA groups across the four treatments, with the FG+SA treatment group positioned in between (Figure 7A and Figure S1). The correlation between physiological characteristics and morphology varies across different tissues. The number of leaves, root diameter, and number of tillers showed a significant positive correlation with the levels of POD, CAT, and H_2_O_2_ in the roots. Conversely, they exhibited a negative correlation with CInv, SS, and VAIV (Figure 7B). The present study examines the correlation between phenotypes and physiological changes in stems, which can be divided into two distinct categories. Firstly, the parameters of PH, FW, RW, and RL demonstrated a positive correlation with hydrogen peroxide levels in stems. Conversely, JA exhibited a positive correlation with NT, RD, and NB. Interestingly, carbohydrate metabolism displayed a negative correlation with phenotypic changes. Moreover, the phenotypic changes demonstrated a negative correlation with the NO and POD (Figure 7C). The phenotypic traits NB, RL, RW, FW, and PH exhibited positive correlations with JA and SA in leaves, while demonstrating negative correlations with carbohydrate metabolism. Additionally, phenotypic changes were found to be negatively correlated with H_2_O_2_ metabolism and SOD activity in the redox balance (Figure 7D).

**Figure 8:**
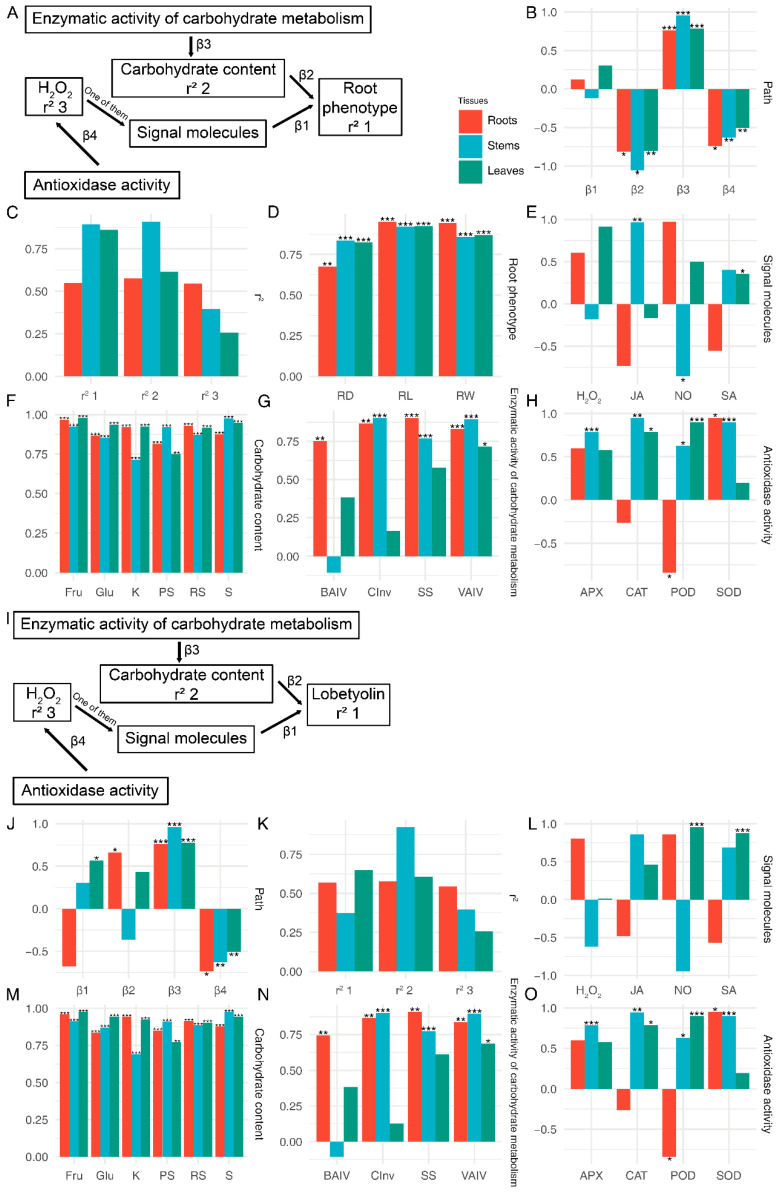
SEM analysis of the relationship between the root phenotype (lobetyolin) and physiological characteristics of *C. pilosula* in three different tissues. (**A**) The PLS-SEM diagram illustrating the relationship between the root phenotype and physiological characteristics. (**B**) The estimates of paths in the root phenotype section. (**C**) The variance-explained r-squared of the PLS-SEM in the root phenotype section. (**D**–**H**) The loading factor between latent variable and manifest variable in the root phenotype section. (**I**) The PLS-SEM diagram illustrating the relationship between the lobetyolin and physiological characteristics. (**J**) The estimates of paths in the lobetyolin. (**K**) The variance-explained r-squared of the PLS-SEM in the lobetyolin. (**L**–**O**) The loading factor between latent variable and manifest variable in the lobetyolin. PH: plant height; FW: fresh weight; RL: root length; RW: root weight; RD: root diameter; NB: the number of leaves; NT: the number of tillers; KS: soluble sugar; RS: reduced sugar; PS: polysaccharide; Glc: glucose; Suc: sucrose; Fru: fructose; SP: soluble protein; BAIV: cell wall acid invertase; CInv: cytoplasmic invertase; VAIV: vacuolar acid invertase; SS: sucrose synthase; NO: nitric oxide; H_2_O_2_: hydrogen peroxide; SA: salicylic acid; JA: jasmonate acid; POD: peroxidase; SOD: superoxide dismutase; CAT: catalase; APX: ascorbate peroxidase; R: root; S: stem; L: leaf. *: *p* < 0.05, **: *p* < 0.01, ***: *p* < 0.001.

The establishment of structural equations between physiological data and phenotypes was conducted using the PLS-SEM algorithm. The study employed the PLS-SEM algorithm to construct structural equations linking physiological data with phenotypes (Figure 8A–H and Figure S2). To assess the reliability of the equations, the relationships between physiological indicators and root morphology, as well as other morphological data, were calculated independently (Figure 8A and Figure S2A). The results of the structural equation analysis revealed that carbohydrate content exerted a negative impact on root length, root diameter, and root weight, while enzymatic activity of carbohydrate metabolism displayed a positive influence on carbohydrate content (Figure 8A). Furthermore, antioxidase-like activity demonstrated a positive effect on H_2_O_2_ (*p* < 0.05). Physiological changes in all three tissues had an impact on root morphological indicators (Figure 8D). Among the root physiological indicators, root length, and weight exhibited the strongest correlation, whereas root diameter showed the strongest correlation with stem physiological data (*p* < 0.05) (Figure 8D). The predominant loading factor for carbohydrate content was fructose in root leaves and sucrose in stems (*p* < 0.05) (Figure 8F). Of the enzymatic activity of carbohydrate metabolism, the highest loading factor was observed for the SS in roots, CInv in stems, and VAIV in leaves (*p* < 0.05) (Figure 8G). With regard to antioxidant-like activity, SOD displayed the highest loading factor in roots, while POD had a negative loading factor (Figure 8H). In stems, CAT demonstrated the highest loading factor, while in leaves, POD exhibited the highest loading factor (Figure 8H).

The PLS-SEM analysis conducted on other morphologies revealed certain distinctions compared to the analysis conducted on roots (Figure S2A). In leaves, signalling molecules exhibited a positive impact on other morphological indicators, whereas changes in carbohydrate content in both leaves and roots displayed a negative effect (*p* < 0.05) (Figure S2B). The findings of other influencing pathways were analogous to those observed in root phenotype (Figure S2B). The physiological index most profoundly affected was pH, whereas FW was primarily influenced by physiological changes in roots and leaves, and NB was predominantly influenced by physiological changes in stems (Figure S2D). The key signalling molecules involved in leaf influence were NO and SA (*p* < 0.05) (Figure S2E).

To elucidate the relationship between primary metabolites and secondary metabolites, we employed PLS-SEM to analyse the correlation between physiological data and lobetyolin. In comparison to phenotypic structures, the fluctuations in carbohydrates positively influence the levels of lobetyolin in both roots and leaves, exerting a significant impact (*p* < 0.05) on the variations of lobetyolin specifically in the roots (Figure 8J). The most pronounced influence on lobetyolin within the leaves is exerted by (Figure 8L). The alterations in the loading factors of carbohydrate content, enzymatic activity of carbohydrate metabolism, and antioxidase-like activity mirror the findings of the root analysis.

## 4. Discussion

A number of studies have indicated that endophytic fungi may play a role in promoting plant growth and enhancing their resistance through interactions with salicylic acid. Some studies have demonstrated that endophytic fungi can induce the production and activation of SA in plants, thereby triggering their defence responses [49]. However, other studies have suggested that exposure to SA may hinder certain functions of these endophytic fungi [50]. The present study demonstrated that the *P. chrysanthemicola* enhances the growth of *C. pilosula*. Furthermore, a comparison was conducted between the outcomes of the *P. chrysanthemicola* and the treatment of exogenous SA, which revealed an antagonistic phenomenon between them. The findings of this study demonstrate that the *P. chrysanthemicola* enhances the biomass accumulation in *C. pilosula*, in contrast to the suppressive impact observed when treating *C. pilosula* with SA. Moreover, the co-treatment of *P. chrysanthemicola* and SA mitigates the inhibitory effect.

The theory of growth–differentiation balance posits that alterations in carbon source supply influence the carbon pool of secondary metabolites, thereby moderating the cost of growth [51]. The invasion of endophytic fungi into plants has the potential to alter the plant’s carbohydrate metabolism, which is further influenced by the regulation of SA on the immune system. Some fungi exhibit a symbiotic relationship with certain plants, during which they modulate carbohydrate metabolism, particularly glucose, sucrose, and starch, by interfering with key metabolic pathways such as glycolysis, thereby impacting the host plant’s energy dynamics [16,17]. SA has been demonstrated to promote the synthesis of glucose and sucrose, while simultaneously inhibiting the tricarboxylic acid cycle (TCA) [52,53,54]. It regulates sucrose metabolism by modulating key enzymes, including invertase and sucrose synthase, thereby providing crucial energy and carbon sources for defence responses [18,19]. The regulation of specific invertases (BAIV, CInv, VAIV) by both *P. chrysanthemicola* and SA plays a crucial role in modulating carbohydrate metabolism [55,56,57,58,59]. This experiment also found a similar phenomenon. The alterations in carbohydrate levels and enzyme activity demonstrate the strain’s capacity to impede the supply of carbon sources and energy for defensive reactions against SA [60]. Carbohydrate catabolism serves as a primary source of carbon and energy for secondary metabolic pathways, enabling the synthesis of a diverse array of end products [61]. The observed relationship highlights a substantial association between the variety of endophytic microorganisms and the content of lobetyolin in *C. pilosula*’s secondary metabolites [20]. The experimental results consistently demonstrate the antagonistic influence of *P. chrysanthemicola* on plant responses to SA. Furthermore, the analysis of lobetyolin, a distinctive secondary metabolite of *C. pilosula*, utilizing content and PLS-SEM, illustrates the strain’s capacity to mitigate the influence of SA on the balance between growth and differentiation. This relationship between carbohydrate metabolism and the observed changes in phenotype and lobetyolin concentrations elucidates the underlying mechanism of this experiment.

Plants possess a sophisticated immune system that enables them to effectively respond to microorganisms. This immune response involves the coordination of multiple signal molecules, forming complex regulatory networks responsible for mediating the synthesis of secondary metabolites [62,63,64]. Infection by endophytic fungi activates cell surface receptors, influencing the behaviour of signalling molecules such as SA, H_2_O_2_, and NO [9]. Through symbiotic relationships with microbes, plants can enhance their defence responses and manipulate the expression of defence genes, a process known as induced systemic resistance (ISR) [65]. SA serves as a key regulator of ISR, playing a crucial role in modulating the production of reactive oxygen species (ROS) in response to stress [66]. Low concentrations of SA stimulate ROS production, while high levels can lead to oxidative stress [67,68]. The combination of endophytes and SA reduces the production of ROS in plants, thereby improving biomass recovery and mitigating the adverse effects caused by osmotic stress [69]. This experiment observed the inhibitory effect of the combined use of *P. chrysanthemicola* and SA on the production of ROS in plant cells. It also identified that using *P. chrysanthemicola* alone enhances the activity of ROS scavenging enzymes, whereas the use of SA alone elevates ROS levels. Consequently, these factors induce alterations in the H_2_O_2_ content within plant cells. H_2_O_2_ is a significant signalling molecule that plays a pivotal role in stress responses and metabolic processes by inducing the expression of stress-tolerance genes [70]. The formation of symbiotic relationships between plants and endophytic fungi results in a reduction of H_2_O_2_ content [71]. The inhibition of root-produced H_2_O_2_ resulting from the application of exogenous SA was successfully alleviated through the combined treatment of *P. chrysanthemicola* and SA. Furthermore, endophytic fungi enhance nodulation and nitrogen fixation by promoting NO signalling, which is dependent on SA [28]. The relationship between SA and NO is significant, as the SA induced increased NO levels under biotic stress [72]. In this experiment, the co-treatment of *P. chrysanthemicola* and SA was found to alleviate the inhibition of leaf NO induced by exogenous SA. These results provide insights into the complex interactions between plants, microorganisms, and signalling molecules, offering valuable insights into their impact on the activity of antioxidant enzymes and the levels of ROS, SA, NO, and H_2_O_2_ in plants.

## 5. Conclusions

The study revealed that in the context of the symbiotic relationship between *C. pilosula* fungi and plants, an antagonistic interplay with salicylic acid is observed. This interaction is particularly evident in the regulation of carbon allocation to secondary metabolism, where the simultaneous involvement of two critical signalling molecules, ROS and NO, is essential for a comprehensive understanding of metabolic dynamics. Future research into the complex relationship between fungi and plants will focus on carbon metabolism, secondary metabolic pathways, and the regulatory role of signalling molecules in orchestrating these processes.

## Data Availability

Genome accession is JARVTL000000000.1. Data will be made available on request.

## Supplementary Materials

The following supporting information can be downloaded at: https://www.mdpi.com/article/10.3390/biology13060408/s1, Figure S1: PLSR analysis of the scores plotted in the results of phenotypic changes versus physiological changes in stems (A) and leaves (B). Figure S2: Structural equation modelling (SEM) analysis of the relationship between the other phenotype and physiological characteristics of *C. pilosula* in three different tissues.
