# Supplementary material for: Paraphoma chrysanthemicola Affects the Carbohydrate and Lobetyolin Metabolism Regulated by Salicylic Acid in the Soilless Cultivation of Codonopsis pilosula
Source: Biology (Basel). 2024 Jun 3;13(6):408. doi: 10.3390/biology13060408 (PMC11200528; doi:10.3390/biology13060408)
Supplement: Supplementary file 1 [file biology-13-00408-s001.zip › biology-2901237-supplementary.pdf]

Supplement figures:

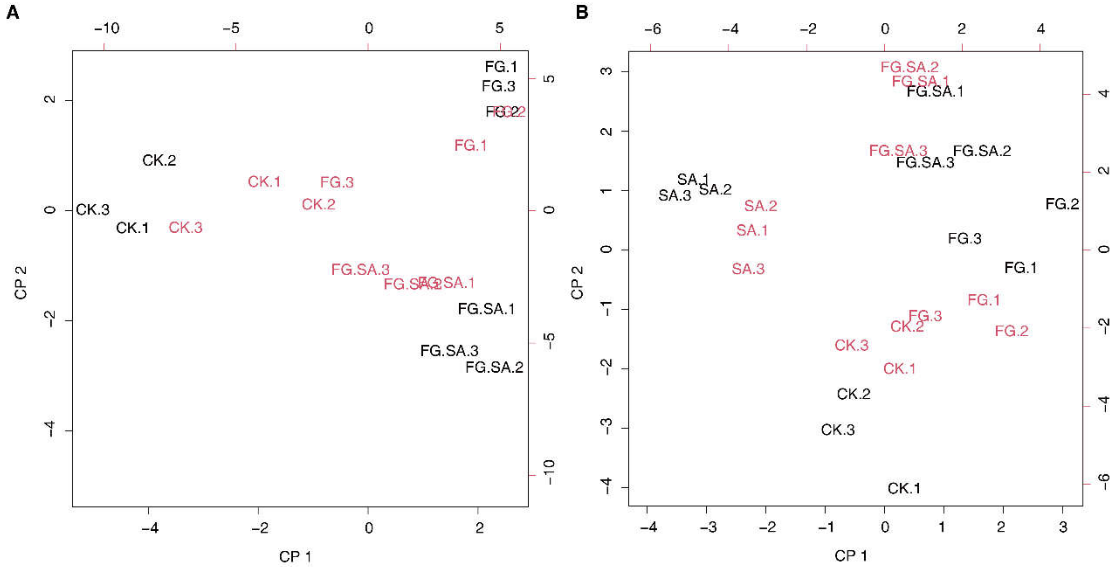

Figure S1 PLSR analysis of the scores plotted in the results of phenotypic changes versus physiological changes in stems (A) and leaves (B). PH: plant height; FW: fresh weight; RL: root length; RW: root weight; RD: root diameter; NB: the number of leaves; NT: the number of tillers; KS: soluble sugar; RS: reduced sugar; PS: polysaccharide; Glc: glucose; Suc: sucrose; Fru: fructose; SP: soluble protein; L: lobetyolin; BAIV: cell wall acid invertase; CInv: cytoplasmic invertase; VAIV: vacuolar acid invertase; SS: sucrose synthethase; NO: nitric oxide; H2O2: hydrogen peroxide; SA: salicylic acid; JA: jasmonate acid; POD: peroxidase; SOD: superoxide dismutase; CAT: catalase; APX: ascorbate peroxidase; R: root; S: stem; L: leaf. The two concentric circles were the default radii corresponding to 50% and 100% explained variance.

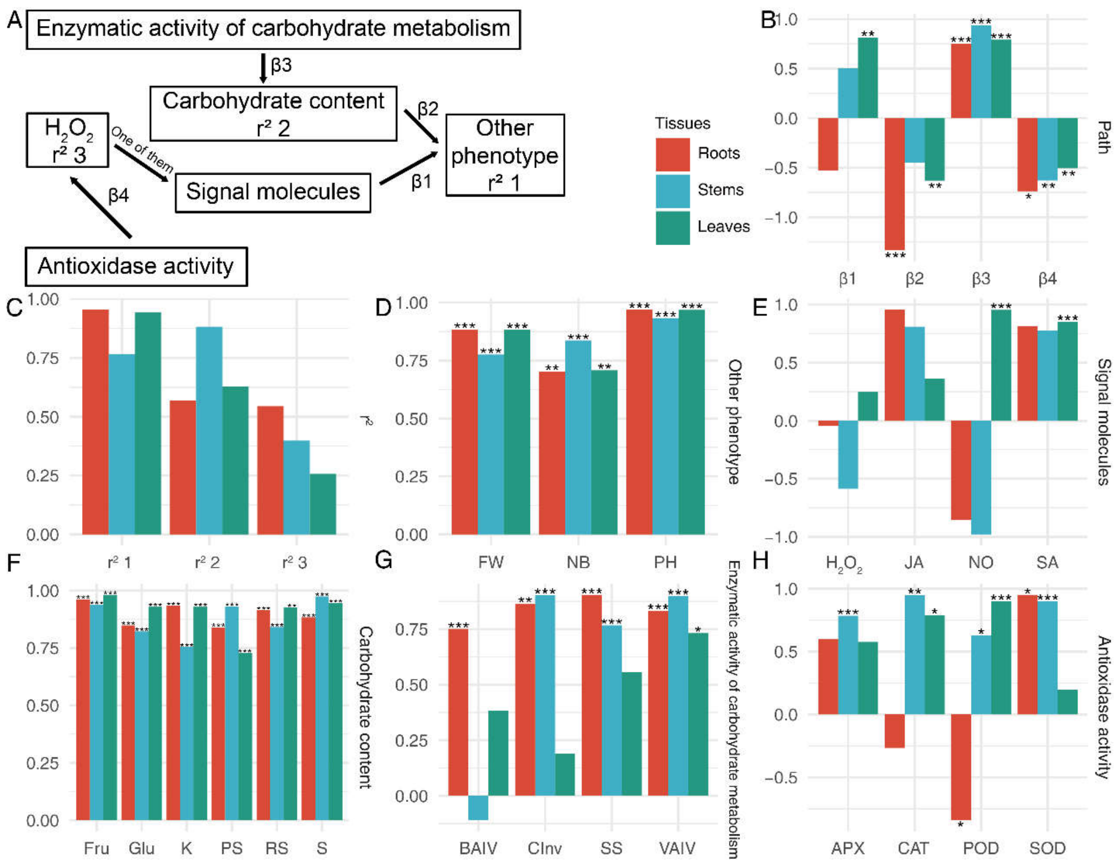

Figure S2 Structural equation modeling (SEM) analysis of the relationship between the other phenotype and physiological characteristics of *C. pilosula* in three different tissues. A: The PLS-SEM diagram illustrating the relationship between the other phenotype and physiological characteristics. B: The estimates of paths in the other phenotype section. C: The variance-explained r-squared of the PLS-SEM in the oother phenotype section. D-H: The loading factor between latent variable and manifest variable in the root phenotype section. PH: plant height; FW: fresh weight; RL: root length; RW: root weight; RD: root diameter; NB: the number of leaves; NT: the number of tillers; KS: soluble sugar; RS: reduced sugar; PS: polysaccharide; Glc: glucose; Suc: sucrose; Fru: fructose; SP: soluble protein; BAIV: cell wall acid invertase; CInv: cytoplasmic invertase; VAIV: vacuolar acid invertase; SS: sucrose synthase; NO: nitric oxide; H2O2: hydrogen peroxide; SA: salicylic acid; JA: jasmonate acid; POD: peroxidase; SOD: superoxide dismutase; CAT: catalase; APX: ascorbate peroxidase; R: root; S: stem; L: leaf. *: p<0.05, **: p<0.01, ***: p<0.001.
